# Encapsulation of Fe_3_O_4_ Nanoparticles into N, S co-Doped Graphene Sheets with Greatly Enhanced Electrochemical Performance

**DOI:** 10.1038/srep27957

**Published:** 2016-06-14

**Authors:** Zunxian Yang, Kun Qian, Jun Lv, Wenhuan Yan, Jiahui Liu, Jingwei Ai, Yuxiang Zhang, Tailiang Guo, Xiongtu Zhou, Sheng Xu, Zaiping Guo

**Affiliations:** 1 National & Local United Engineering Laboratory of Flat Panel Display Technology, Fuzhou University, Fuzhou 350002, P. R. China; 2 Institute for Superconducting & Electronic Materials, University of Wollongong, NSW 2522, Australia; 3 School of Mechanical, Materials & Mechatronics Engineering, University of Wollongong, NSW 2522, Australia

## Abstract

Particular N, S co-doped graphene/Fe_3_O_4_ hybrids have been successfully synthesized by the combination of a simple hydrothermal process and a subsequent carbonization heat treatment. The nanostructures exhibit a unique composite architecture, with uniformly dispersed Fe_3_O_4_ nanoparticles and N, S co-doped graphene encapsulant. The particular porous characteristics with many meso/micro holes/pores, the highly conductive N, S co-doped graphene, as well as the encapsulating N, S co-doped graphene with the high-level nitrogen and sulfur doping, lead to excellent electrochemical performance of the electrode. The N-S-G/Fe_3_O_4_ composite electrode exhibits a high initial reversible capacity of 1362.2 mAhg^−1^, a high reversible specific capacity of 1055.20 mAhg^−1^ after 100 cycles, and excellent cycling stability and rate capability, with specific capacity of 556.69 mAhg^−1^ when cycled at the current density of 1000 mAg^−1^, indicating that the N-S-G/Fe_3_O_4_ composite is a promising anode candidate for Li-ion batteries.

The emergence of electric vehicles (**EV**) and hybrid electric vehicles (**HEV**) and the popularity of portable electronics, as well as the applications of the power grid, trigger the requirement of rechargeable batteries with both high-energy capacity and power density. Of all the energy storage devices, lithium ion batteries (**LIBs**), have attracted more and more attention mainly due to their most possibilities in meeting the requirements for the high energy equipment including electric vehicles (**EV**), hybrid electric vehicles (**HEV**) and the power grid^1^,^2^,^3^,^4^,^5^,^6^. The suitable nanomaterials and nanostructures that allow fast charge-discharge rate capability would be one of the most important respects to the high-performance lithium ion batteries. Many electrode materials, especially anode materials, such as Si^7^, SnO_2_^8^,^9^, CuO^10^ and some other transition metal oxides, have been investigated and used for the lithium ion battery. Of all those anode materials mentioned-above, Fe_3_O_4_ has absorbed great interests to many researchers on the lithium ion battery, mainly due to its particular advantages^1^,^11^,^12^ of nature’s abundance, environmental benignity, high electronic conductivity, low cost and much higher storage capacity (Fe_3_O_4_: 926 mA h g^−1^) as compared with the traditional carbonaceous anode materials (Graphite: 372 mA h g^−1^). The practical application of Fe_3_O_4_ in lithium-ion batteries, however, is greatly hampered by its poor cycling performance, as well as its vulnerability to agglomeration and mechanical strain originating from the large volume variation during the lithiation/delithiation processes, which results in severe loss of capacity, increased diffusion lengths and decreased electrical conductivity^13^,^14^,^15^,^16^.

During the past decades, tremendous efforts have been made to overcome these problems mentioned above. One efficient strategy is to design the nanomaterials or nanostructures via reducing the particle size of Fe_3_O_4_ down to the nanometer scale, which could accommodate or buffer the volume changes, greatly reduce the strain that originates from the lithiation and dilithiation process, directly shorten the transport path and the diffusion time for lithium ions, and furthermore, offer more active sites for lithium ions during charge/discharge cycling processes. Many nano-scale Fe_3_O_4_ architectures, such as nanotubes^16^,^17^,^18^, nanobelts^19^, nanofibers^20^,^21^, nanospheres^22^,^23^, nanorods^24^,^25^, and so on, have been prepared by many different methods, including the hydrothermal method^26^, the solvothermal route^27^, the electrospinning method^21^, electrochemical techniques, etc. Another alternative strategy is to combine Fe_3_O_4_ in the form of nanostructures with a high conductivity matrix including various metal nanostructures^1^,^28^, carbon materials^27^,^29^ and other stable materials, which could cushion the mechanical effects aroused during the charge/discharge process and simultaneously improve the conductivity of the composite. Up to now, hybridization of carbon materials including the amorphous carbon^20^, carbon nanotube^16^,^30^, and the recently-developed graphene with Fe_3_O_4_ may be one of the most effective solution. Especially, graphene, as one of carbon materials with honeycomb crystal lattice and one-atom thick planar characteristics, have provided great opportunities in enhancing the performance of Fe_3_O_4_ as the LIB electrodes^31^,^32^,^33^ owing to its excellent electrical conductivity, high mechanical flexibility, large specific surface area, and pronounced thermal and chemical stability. Recently, introducing heteroatoms such as N, B and S etc. into graphene can effectively enhance the lithium storage capacity of graphene-based composite^34^,^35^ mainly owing to greatly increased charged sites and conductivity in the heteroatom doped graphene^36^, which would greatly mitigate the negative effect of graphene content on the lithium ion storage capacity of composite. However, at present, the heteroatom doped graphene/Fe_3_O_4_ composites are still a long way from being ideal anode candidates for LIBs mainly because there are many challenges in forming bi-heteroatom doped graphene/Fe_3_O_4_ composites and enhancing the content of heteroatom doped in the composite.

In this paper, a simple and low-cost strategy is reported to build novel N, S co-doped graphene/Fe_3_O_4_ architectures with Fe_3_O_4_ nanoparticles encapsulated into the N, S co-doped graphene sheets by a hydrothermal process, followed by a subsequent carbonization treatment. Apart from combining the advantages of nanoscale Fe_3_O_4_ particles with those of N, S co-doped graphene, the novel N, S co-doped graphene/Fe_3_O_4_ nanostructure has many unique advantages such as particular bi-heteroatom-doped graphene/Fe_3_O_4_ nanoarchitecture, meso/micro porosities surrounded by many randomly-aligned N, S co-doped graphene nanosheets, large surface-to-volume ratio, and excellent conductivity including both ionic conductivity and electronic conductivity mainly owing to the highly conductive N, S co-doped graphene nanosheets, as well as the high heteroatom-content in the N, S co-doped graphene/Fe_3_O_4_ nanostructures. The N, S co-doped graphene/Fe_3_O_4_ nanostructures have been investigated in a preliminary way for potential use as an anode material for the lithium ion battery and have exhibited excellent cycling stability and rate capability.

## Results

**Figure 1:**
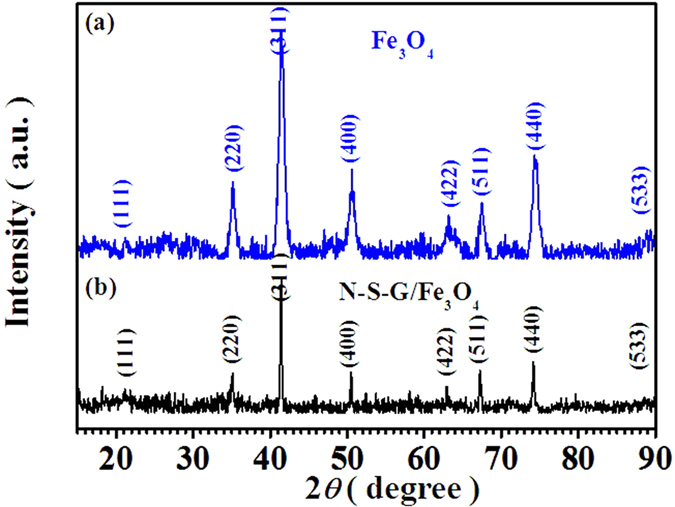
X-ray diffraction patterns of as-prepared Fe_3_O_4_ nanoparticles and N-S-G/Fe_3_O_4_ composite, respectively. (**a**) Fe_3_O_4_ nanoparticles with cubic structure (JCPDS 19-0629), (**b**) N-S-G/Fe_3_O_4_ composite with cubic structure (JCPDS 19-0629), as indexed in the patterns.

**Figure 2:**
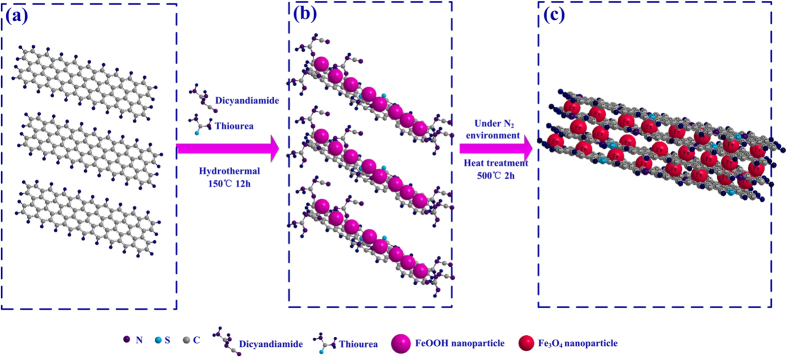
The growth mechanism of Nitrogen and Sulfur co-doped graphene/Fe_3_O_4_ composite: (**a**) graphene sheets, (**b**) formation of N,S co-decorated graphene/FeOOH nanoparticles composite after hydrothermal reaction, (**c**) formation of N, S co-doped graphene/Fe_3_O_4_ composite after heat treatment in N_2_ atmosphere.

**Figure 3:**
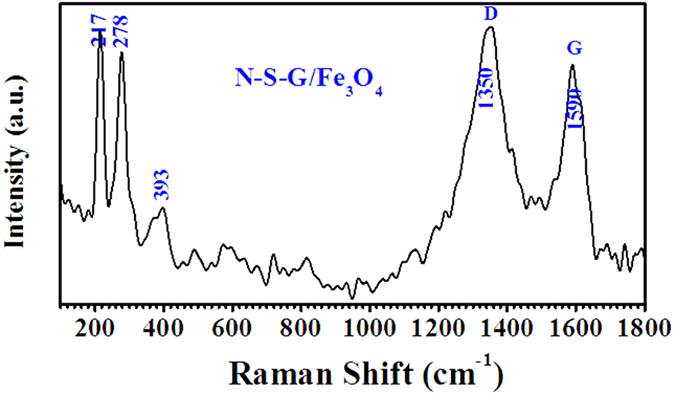
Raman spectra for the as-synthesized N-S-G/Fe_3_O_4_ composite.

The X-ray diffraction patterns of as-prepared Fe_3_O_4_ nanoparticles and N, S co-doped graphene/Fe_3_O_4_ (N-S-G/Fe_3_O_4_) composites, as shown in Fig. 1, reveal that the nanopowders synthesized by simple **LPR** process via just controlling the mole ratio of Fe^3+^ to Fe^2+^ in the precursor solution belong to typical Fe_3_O_4_ with cubic spinel structure (JCPDS 19–0629)^1^,^29^,^37^ according to the obvious reflections from their characteristic lattice planes (220), (311), (400), (422), (511) and (440) (see Fig. 1(a)). When dicyandiamide and thiourea, as well as iron chloride, were dispersed into the GO suspension in turn and experienced hydrothermal process, as illustrated in Fig. 2(a,b), the iron chloride was hydrolyzed to form FeOOH particles^38^,^39^ embedded and grown on graphene surface, and simultaneously, Nitrogen and Sulfur species were incorporated into the graphene lattices during the hydrothermal reaction between dicyandiamide or thiourea and hydroxyl (-OH) or carboxyl (-COOH) in graphene sheets, respectively. Subsequently, after dehydration reaction in 500 °C nitrogen environment, the FeOOH particles were finally transferred to Fe_3_O_4_ nanoparticles encapsulated into N, S co-doped graphene nanosheets (see Fig. 2(c)). As shown in Fig. 1(b), thenanostructuresN-S-G/Fe_3_O_4_ nanostructures is also ascribed to typical Fe_3_O_4_ phase with cubic spinel structure (JCPDS 19–0629), which is in good accordance with that of the pure Fe_3_O_4_ sample, and no other diffraction peaks of graphene and N, S co-doped graphene can be found (see Fig. 1(b)). Raman spectroscopic analysis was further employed to explore the composition of the N-S-G/Fe_3_O_4_ nanostructures. As depicted in Fig. 3, the scattering peaks at 217, 278, 393 cm^−1^ are mainly attributable to the E_g_, T_2g_, and A_1g_ vibration modes of α-Fe_2_O_3_ which possibly originates from the decomposition of Fe_3_O_4_ in the composite under the strong laser light radiation during the Raman measurement^40^. The remaining two characteristic peaks at 1350 and 1590 cm^−1^ are in good agreement with the typical D band and G band^41^,^42^, respectively, which are possibly associated with disordered carbon and the first-order scattering of the E_2g_ mode of sp^2^ carbon domains^43^. Here, the dicyandiamide and thiourea hydrothermal reaction and subsequent dehydration heat-treatment processes obviously facilitate the FeOOH particle uniformly attached on graphene^38^,^39^, Nitrogen and Sulfur co-incorporated graphene, and finally the Fe_3_O_4_ nanoparticle encapsulated into N, S co-doped graphene nanosheets. Those N-S-G/Fe_3_O_4_ nanostructures, consisting of uniformly dispersed Fe_3_O_4_ nanoparticles and the encapsulating N, S co-doped graphene nanosheets with excellent conductivity, effectively combine the advantages of nanoscale Fe_3_O_4_ particles with those of N, S co-doped graphene mainly due to the particular bi-heteroatom-doped graphene/Fe_3_O_4_ nanoarchitecture, the uniformly dispersed Fe_3_O_4_ nanoparticles and the encapsulating N, S co-doped graphene nanosheets.

**Figure 4:**
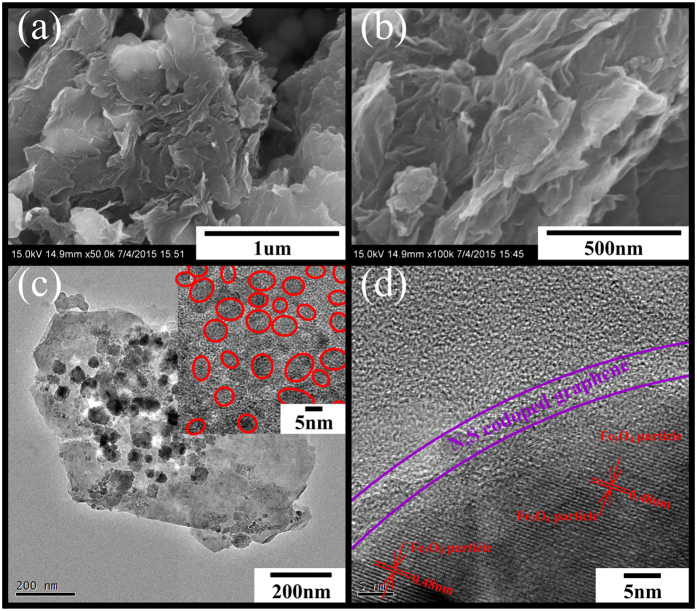
FE-SEM and TEM images of as-prepared N-S-G/Fe_3_O_4_ composite: (**a**) FE-SEM image of as-prepared N-S-G/Fe_3_O_4_ composite revealing the Fe_3_O_4_ nanoparticles encapsulated in the N, S co-doped graphene nanosheets; (**b**) high-magnification FE-SEM image of as-prepared N-S-G/Fe_3_O_4_ composite; (**c**) TEM image of as-prepared N-S-G/Fe_3_O_4_ composite indicating that there are many Fe_3_O_4_ nanoparticles distributed and encapsulated in the N, S co-doped graphene nanosheets (Fe_3_O_4_ nanoparticles with average diameters of ~5–10 nm are dispersed uniformly in in the N, S co-doped graphene nanosheets, as shown in the Inset); (**d**) high magnification TEM image of as-prepared N-S-G/Fe_3_O_4_ composite indicating that the Fe_3_O_4_ nanoparticles are effectively encapsulated into the N, S co-doped graphene nanosheets.

The morphologies of the as-prepared graphene, N-S-G/Fe_3_O_4_ composite, as well as the pure Fe_3_O_4_, have been respectively investigated by the scanning electron microscope (**SEM**), the field-emission scanning electron microscopy (**FE-SEM**) and the Transmission electron microscopy (**TEM**) (see Supporting Information Figure S1 and Fig. 4). As shown in Supporting Information Figure S1(a), the as-prepared graphene nonosheets are randomly stacked up to form fluffy matrix, where there are some wrinkles on the surface of graphene sheets and many meso- or micro- holes/pores in the graphene matrix possibly owing to the rolling up and surrounding effect of graphene planes. Similar phenomenon appears in the as-prepared N-S-G/Fe_3_O_4_ composite, and there are also many wrinkles on their surface and many meso- or micro- holes/pores in the composite except for many Fe_3_O_4_ nanoparticles obviously dispersed on the surface of N, S co-doped graphene nanosheets (see Supporting Information Figure S1(b)). As depicted in Fig. 4(a,b), there are many Fe_3_O_4_ nanoparticles or nanoparticle aggregates encapsulated into the N, S co-doped graphene nanosheets, which not only effectively buffer agglomeration and mechanical strain originating from the large volume variation during the lithiation/delithiation processes but also greatly enhance the conductivity of the composite including both lithium ion conductivity and electron conductivity. In Supporting Information Figure S1(c,d) and Fig. 4(c,d), the TEM images of the as-prepared N-S-G/Fe_3_O_4_ composite have further shed light on their structural and morphological characteristics. There are many Fe_3_O_4_ nanoparticle aggregates uniformly dispersed on the surface of N, S co-doped graphene nanosheets (see Fig. 4(c) and Supporting Information Figure S1(c,d)), and those Fe_3_O_4_ nanoparticle aggregates with diameters of ~80–100 nm actually consist of some tiny Fe_3_O_4_ nanoparticles with diameters of ~5–10 nm aggregated together, respectively (see Fig. 4(c) inset), which are obviously much smaller than the sizes of those pure Fe_3_O_4_ nanoparticles or nanoparticle aggregates (see Supporting Information Figure S1(e,f)). As shown in Fig. 4(d), the high resolution transmission electron microscope (**HRTEM**) image of the N-S-G/Fe_3_O_4_ composite further demonstrates that the Fe_3_O_4_ in the N-S-G/Fe_3_O_4_ composite consists of many nanoparticles/nanocrystals that are uniformly and tightly attached to the surfaces of the N, S co-doped graphene nanosheets to form an encapsulating N-S-G/Fe_3_O_4_ nanoparticles architecture, except that there are a few Fe_3_O_4_ nanocrystals existed in an aggregate form. In addition, the HRTEM image clarifies the crystal structure with an interplanar spacing of approximately 0.480nm between neighboring (111) planes of the cubic spinel Fe_3_O_4_, as those planes were parallel to the electron beam, which is similar to what has been reported previously^44^.

**Figure 5:**
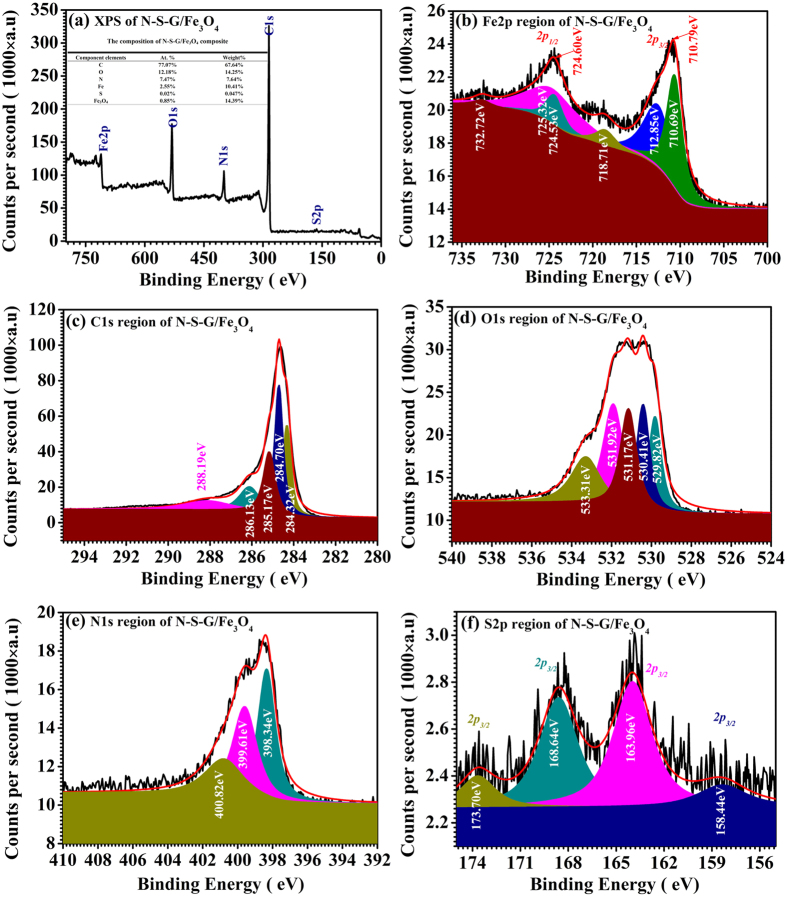
The XPS spectra of N-S-G/Fe_3_O_4_ composite. (**a**) Survey spectrum; (**b**) XPS high-resolution spectra of the Fe2p region; (**c**) XPS high-resolution spectra of the C1s region; (**d**) XPS high-resolution spectra of the O1s region; (**e**) XPS high-resolution spectra of the N1s region; (**f**) XPS high-resolution spectra of the S2p region.

As depicted in Fig. 5, Supporting Information Figures S2 and S3, the X-ray photoelectron spectroscopy (**XPS**) analysis, the element mapping and the energy dispersive X-ray spectroscopy (**EDX**) analysis of the N-S-G/Fe_3_O_4_ composite, as well as the thermogravimetric (**TGA**) and different scanning calorimetric (**DSC**) analysis of the N-S-G/Fe_3_O_4_ composite, have been conducted to further explore the composition of the N-S-G/Fe_3_O_4_ composite, respectively. The XPS analysis of the N-S-G/Fe_3_O_4_ composite not only clearly demonstrates the presence of Fe, C, O, N and S elements (see Fig. 5(a)) but also discloses the chemical compositions and chemical oxidation states of the Fe, C, O, N and S elements in the N-S-G/Fe_3_O_4_ composite via fitting their own corresponding **XPS** high-resolution spectra of the Fe2p region, C1s region, O1s region, N1s region and S2p region (see Fig. 5(a) inset and Fig. 5(b–f)), respectively, which is in good accordance with its elemental mapping and EDX result (see Figure S2). The Fe2p spectrum (see Fig. 5(b)) for the N-S-G/Fe_3_O_4_ composite consists of two symmetrical broadened peaks with the binding energies (**BE**s) of 710.79 eV and 724.60 eV, which are attributable to the Fe2p3/2 and Fe2p1/2, respectively, for both Fe^2+^ and Fe^3+^ ions in the Fe_3_O_4_, actually the mixed state of FeO and Fe_2_O_3_. These values agree very well with the literature values^45^. And the two small satellite peaks of Fe 2p3/2 and Fe2p1/2 at 718.71 eV and 732.72 eV (see Fig. 5(b)) in the N-S-G/Fe_3_O_4_ composite further reveal the presence of a very small amount of g-Fe_2_O_3_^45^. The XPS pattern of Fe2p effectively reveals the existence of iron element in the form of Fe_3_O_4_ for our N-S-G/Fe_3_O_4_ composite, which is in good agreement with the XRD result of the N-S-G/Fe_3_O_4_ composite (see Fig. 1(b)). As shown in Fig. 5(a) inset, the calculated Fe_3_O_4_ content in the composite is 14.39wt%, agreeing well with what is obtained from the **EDX** analysis result of the N-S-G/Fe_3_O_4_ composite (14.825wt%, Figure S2(h) inset), but much smaller than that from the TGA result (53.37wt%, Figure S3). The larger difference between the calculated Fe_3_O_4_ content in the composite according to the XPS and the EDX result and that derived from the TGA analysis is possibly due to the cause that the former two means belong to one of surface-testing techniques on material composition while the latter is one of bulk phase-testing techniques. In other words, most of Fe_3_O_4_ in the N-S-G/Fe_3_O_4_ composite exists in the form of Fe_3_O_4_ nanoparticles encapsulated into the N, S co-doped graphene nanosheets, which are in good accordance with the SEM, FE-SEM and TEM results (see Fig. 4 and Figure S1). As for the C1s spectrum in the N-S-G/Fe_3_O_4_ composite (see Fig. 5(c)), the two strongest peaks at 284.70 eV and 284.32 eV are attributed to the graphitic carbon in un-oxidized graphitic carbon matrix, whereas the following peak at 285.17 eV is partly attributed to the existence of some nitrogen-containing functional group in organic matrix (C=N) after heat treatment under relative low temperature^46^,^47^, which is in good accordance with the Nitrogen element mapping result (see Figure S2(e)). The remaining two small peaks at 286.10 eV and 288.19 eV possibly come from a trace amount of carboxyl in the composite sample^46^,^47^. As shown in Fig. 5(d), the main portion of the O1s spectrum response could come from the Fe-O bond in the N-S-G/Fe_3_O_4_ composite, as evidenced by O1s binding energy (BE) peak at ~529.82 eV and 530.41 eV (see Fig. 5(d)), which probably arises from the mixed state nature of FeO and Fe_2_O_3_ for Fe_3_O_4_ in the composite^45^. And those peaks at 531.17 eV and 531.13 eV may be due to the OH^−^ radical, adsorbed oxygen, or carbonyl^47^. As for the high BE peaks at 533.31 eV (Fig. 5(d)), it possibly originates from the absorbed H_2_O outside^47^. As shown in Fig. 5(a) inset, the calculated N content in the sample is 7.64 at%, much higher than the EDX result of nitrogen in composite (2.41%). And the main fitted N1s peaks at 398.34 eV and 399.16 eV (see Fig. 5(e)) represent pyridinic and pyrrolic types of N atoms in hybrids^46^,^47^, respectively, while the remaining fitted N1s peak at 400.82 eV (see Fig. 5(e)) possibly originates from the nitrogen oxide (N-O bond) in the composite^47^. The high-level nitrogen doping and pyridinic-like substructures can provide a feasible pathway for Li^+^ penetration into the graphene-layers, which is beneficial for enhancing the electrochemical performances^34^,^48^. The weak S2p spectrum (see Fig. 5(f)) for the N-S-G/Fe_3_O_4_ composite mainly consists of two peaks with the binding energies (**BE**s) of 163.96 eV and 168.64 eV, which are probably attributable to the S2p 3/2 of carbon sulfide (C-S bond) and that of S, O-contained composite (S-O bond)^47^,^49^,^50^, respectively, while the remaining two satellite peaks with the binding energies (**BE**s) of 158.44 eV and 173.07 eV are probably attributable to the S2p3/2 of little iron sulfide and that of sulfur oxide^47^,^49^,^50^, respectively. From a combination of the XRD, Raman, SEM, FE-SEM and TEM results, as well as the XPS results, it is concluded that the N-S-G/Fe_3_O_4_ composite mainly consists of many uniformly dispersed Fe_3_O_4_ nanoparticles/nanocrystals and the N, S co-doped graphene nanosheets. The Fe_3_O_4_ nanoparticle/nanocrystals, together with the N, S co-doped graphene nanosheets, effectively form a particular encapsulating N.S co-doped graphene nanosheets/Fe_3_O_4_ nanoparticles architecture. In addition, there are many meso/micro- holes/voids/pores surrounded by many randomly-aligned N, S co-doped graphene nanosheets. The particular encapsulating N, S co-doped graphene nanosheets/Fe_3_O_4_ nanoparticles architecture would possess many unique advantages in lithium ion battery application, such as high conductivity owing to encapsulation of Fe_3_O_4_ nanoparticles/nanocrystals into the N, S co-doped graphene nanosheets with high conductivity, improved Li^+^ and electrolyte transport in electrode material because of many meso/micro- holes/voids/pores surrounded by many randomly-aligned N, S co-doped graphene nanosheets, enhanced lithium intercalation sites owing to the high-level nitrogen and sulfur doping for the composite and so on, all of which would greatly enhance the electrochemical performance of the electrode.

**Figure 6:**
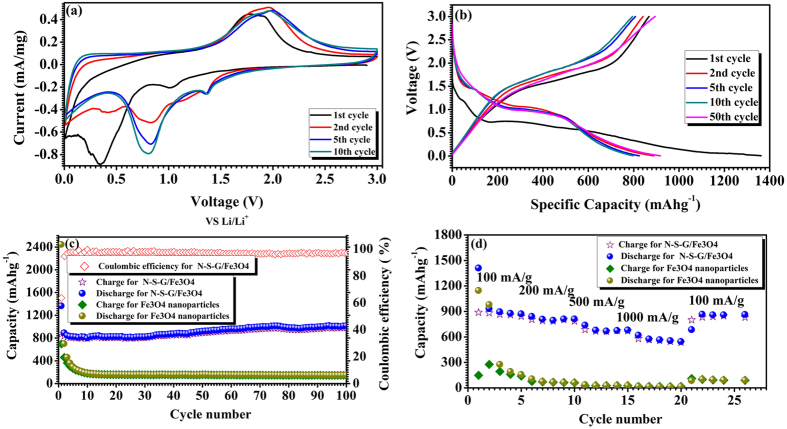
Electrochemical performance of N-S-G/Fe_3_O_4_ composite and Fe_3_O_4_ nanoparticles electrodes cycled between 0.01 and 3.0 V vs. Li^+^/Li. (**a**) Cyclic voltammograms of N-S-G/Fe_3_O_4_ composite electrode of the 1^st^, 2^nd^, 5^th^ and 10^th^ cycles at a scan rate of 0.1 mVs^−1^ in the voltage range of 0.01–3.0 V. (**b**) Voltage profiles for selected cycles of N-S-G/Fe_3_O_4_ composite electrode at the current density of 100 mAg^−1^. (**c**) Capacity vs. cycle number curves and coulombic efficiency from the first cycle to the 100^th^ cycle for the N-S-G/Fe_3_O_4_ composite and Fe_3_O_4_ nanoparticles at the current density of 100 mAg^−1^ with cut-off voltage between 0.01 and 3.0 V. (**d**) Rate capabilities of N-S-G/Fe_3_O_4_ composite and Fe_3_O_4_ nanoparticles electrodes at various currents (100 mA/g, 200 mA/g, 500 mA/g, 1000 mA/g).

The electrochemical performances of the N-S-G/Fe_3_O_4_, reduced graphene oxide/Fe_3_O_4_ (rGO/Fe_3_O_4_), N, S co-doped graphene/Fe_3_O_4_ composites with low content of Fe_3_O_4_ (L-N-S-G/Fe_3_O_4_), N, S co-doped graphene/Fe_3_O_4_ composites with high content of Fe_3_O_4_ (H-N-S-G/Fe_3_O_4_) and Fe_3_O_4_ nanoparticles electrodes including the galvanostatic discharge-charge cycling and Cyclic Voltammetry have systematically been evaluated (see Fig. 6 and Supporting Information Figure S4). The cyclic voltammograms (**CV**s) of the N-S-G/Fe_3_O_4_ composite electrode including the 1^st^, 2^nd^, 5^th^ and 10^th^ cycles at a scan rate of 0.1 mVs^−1^ with the cutoff voltages between 0.01 and 3.0 V were obtained and are plotted in Fig. 6(a), respectively. The curves of the initial few cycles are different from the later ones, especially with respect to the rapid disappearance of the obvious cathodic peak at 0.3447 V, which is probably ascribed to the conversion reaction that results in the formation of Li_2_O and iron^51^,^52^. In the subsequent cycles, this peak shifts to higher voltage 0.8218 V, indicating that there is some structural variation in the N-S-G/Fe_3_O_4_ composite electrode after lithium ion insertion in the first cycle. This is further evidenced by the **CV** result of the pure Fe_3_O_4_ electrode (see Figure S4(a)) as well as that of rGO/Fe_3_O_4_ composite (Figure S4(b)). As shown in Figure S4(a), the cathodic peak at 0.2838 V in the first cycle shifts to higher voltage 0.6255 V in the subsequent cycles, which is also possibly attributable to the structural variation in the pure Fe_3_O_4_ composite electrode after lithium ion insertion in the first cycle. The similar phonomenon observed in the rGO/Fe_3_O_4_ composite sample (Figure S4(b)) except that the cathodic peak at 0.4323 V in the first cycle shifts to higher voltage 0.7097 V in the subsequent cycles. And the obvious decrease of peak intensity reveals the irreversible lithium ion capacity loss possibly owing to the formation of an inactive solid-electrolyte interphase (**SEI**) during the first cycle^51^,^52^,^53^,^54^. From the fifth cycle, stable cathodic/anodic peak pair at 0.8218 V and 1.9 V in the N-S-G/Fe_3_O_4_ composite electrode (Fig. 6(a)) and that at 0.6255 V and 1.5653 V in the pure Fe_3_O_4_ electrode (Figure S4(a)), as well as that at 0.7097 V and 1.8314 V in the rGO/Fe_3_O_4_ electrode (Figure S4(b)), belong to the characteristic cathodic/anodic peak pair of the Fe_3_O_4_-based electrode^51^,^52^,^53^,^54^. Additionally, with the increase of the cycle number, there is a slight shift of the cathodic/anodic peak pair in the N-S-G/Fe_3_O_4_ composite electrode (Fig. 6(a)) and in the rGO/Fe_3_O_4_ composite electrode, which is probably ascribed to the superposition effect of the characteristic cathodic/anodic peak pair from the N, S co-doped graphene and reduced graphene and that from Fe_3_O_4_ in composite.

The cycling performance of the N-S-G/Fe_3_O_4_, rGO/Fe_3_O_4_, L-N-S-G/Fe_3_O_4_, H-N-S-G/Fe_3_O_4_ composite electrodes, as well as that of Fe_3_O_4_ nanoparticles electrode were further probed in the voltage range of 3.0–0.01 V (vs. Li/Li^+^) at a constant current density of approximately 100 mAg^−1^ up to 100 cycles. Figure 6(b) reveals the voltage profiles of N-S-G/Fe_3_O_4_ composite electrode at the current density of 100 mAg^−1^. The first discharge and charge steps deliver a specific capacity of 1362.2 and 867.3 mAh/g, respectively, while the pure Fe_3_O_4_ and rGO/Fe_3_O_4_ composites electrode does the 2443.1 and 696.1 mAhg^−1^, 1585.7 and 987.4 mAhg^−1^ in the first discharge and charge steps (see Figure S4(c,d)). The initial coulombic efficiency of the N-S-G/Fe_3_O_4_ composite electrode is above 63.6%, greatly higher than that of the pure Fe_3_O_4_ electrode (28.7%) and some slightly higher than that of rGO/Fe_3_O_4_ composites electrode (62.2%), which is possibly due to the encapsulation of the N, S co-doped graphene nanosheets and their conductivity enhancement in lithium ions and electrons^8^,^55^. Figure 6(c) indicates the curves of the charge/discharge capacity versus the cycle number and the coulombic efficiency for the N-S-G/Fe_3_O_4_ composite electrode and pure Fe_3_O_4_ electrode at the current density of 100 mAg^−1^, respectively. The N-S-G/Fe_3_O_4_ composite electrode exhibits excellent cycling performance and a high reversible specific capacity of over 854 mAhg^−1^ after the first 10 cycles. Moreover, it rises up slowly to a reversible capacity of approximately 1034.33 mAhg^−1^ after 70 cycles and maintains a reversible capacity of approximately 1055.20 mAhg^−1^ after 100 cycles with the coulombic efficiency of nearly 100%, not only much higher than the specific capacity of pure Fe_3_O_4_ nanoparticles (148.38 mAhg^−1^, see Fig. 6(c)) after 100 cycles, but also higher than those of the rGO/Fe_3_O_4_ (758.5mAhg^−1^, see Figure S4(e)), L-N-S-G/Fe_3_O_4_ (568.2mAhg^−1^, see Figure S4(f)), H-N-S-G/Fe_3_O_4_ (581.6mAhg^−1^, see Figure S4(f)) composite. When compared with that for pure Fe_3_O_4_ electrode, the greatly enhancement in the lithium ion storage capacity for the N-S-G/Fe_3_O_4_ composite electrode is mainly attributed to the short lithium ion diffusion paths, easy access of the electrolyte through the meso/micro-pores surrounded by the randomly-aligned N, S co-doped graphene nanosheets, as well as the good electrical connectivity. As compared with the rGO/Fe_3_O_4_, L-N-S-G/Fe_3_O_4_ and H-N-S-G/Fe_3_O_4_, respectively, the greatly enhancement in the lithium ion storage capacity for the N-S-G/Fe_3_O_4_ composite electrode is possibly attributed to nature of N, S co-doped graphene nanosheets with good electrical conductivity and high lithium ion storage, an appropriate amount of Fe_3_O_4_ nanoparticles in the composite and fully encapsulated Fe_3_O_4_ nanoparticles into N, S co-doped graphene nanosheets, respectively, which all together results in high lithium ion storage capacity for the N-S-G/Fe_3_O_4_ composite electrode. As demonstrated in Fig. 6(d), excellent rate performance has been acquired for the N-S-G/Fe_3_O_4_ composite electrode. The composite electrode delivers a discharge capacity of over 876.2 mAhg^−1^ at the current density of 100 mAg^−1^, 820.31 mAhg^−1^ at 200 mAg^−1^, 688.87 mAhg^−1^ at 500 mAg^−1^, 556.69 mAhg^−1^ at 1000 mAg^−1^, respectively, finally recovers to around 886.02 mAhg^−1^ when the current density goes back to 100 mAg^−1^, which is not only much better than that for the pure Fe_3_O_4_ electrode, but also than those of the rGO/Fe_3_O_4_ (Figure S1(g)), L-N-S-G/Fe_3_O_4_ and H-N-S-G/Fe_3_O_4_ (Figure S2(h)) composite electrodes. In order to make clear the electrochemical nature of the N-S-G/Fe_3_O_4_ composite and pure Fe_3_O_4_ electrodes, as depicted in Figure S5, the electrochemical impedance spectroscopy (**EIS**) patterns of the N-S-G/Fe_3_O_4_ composite electrode, as well as that of the pure Fe_3_O_4_ electrode, were obtained in the frequency range 100 kHz to 0.01 Hz with an amplitude of 10 mV. Additionally, the table of the fitted kinetic parameters was inserted into the Nyquist plots. As compared with those of the pure Fe_3_O_4_ electrode, the smaller Rel1 and Rsl1 of the N-S-G/Fe_3_O_4_ composite electrode represent its smaller ohmic resistance of the battery cell, resistance of SEI film for the N-S-G/Fe_3_O_4_ composite electrode, respectively. Therefore, the great enhancement of coulombic efficiency, reversible discharge capacity, and rate capability for the N-S-G/Fe_3_O_4_ composite electrode is probably attributed to the nanosize-encapsulation of Fe_3_O_4_ nanoparticles/nanocrystals in the composite and the porous nature (see Fig. 3), as well as the high conductivity owing to N, S co-doped graphene nanosheets distributing uniformly in the composite.

## Discussion

The charge diffusion mechanism of the N-S-G/Fe_3_O_4_ composite electrode is depicted in Figure S6 (Supporting Information). Many Fe_3_O_4_ nanoparticles encapsulated into the N, S co-doped graphene nanosheets are uniformly attached on the surface of the N, S co-doped graphene nanosheets and form a particular architecture with uniformly dispersed Fe_3_O_4_ nanoparticles and N.S co-doped graphene encapsulant. Many meso/micro-pores were surrounded by many randomly-aligned N, S co-doped graphene nanosheets. This particular architecture with Fe_3_O_4_ nanoparticles and N.S co-doped graphene encapsulant, the porous nature with many meso/micro- pores surrounded by randomly-aligned N, S co-doped graphene nanosheets, and the high specific surface area effectively enlarges the electrode-electrolyte contact, shorten the lithium ion diffusion pathways during the discharge/charge cycling and greatly enhance the lithium storage capacity and rate capability. Additionally, the N, S co-doped graphene encapsulant doped with high-content heteroatom in the N-S-G/Fe_3_O_4_ composite would not only greatly enhance the conductivity of the active material during the lithium intercalation/de-intercalation but also increase the charged sites and the lithium capacity^34^,^35^, which plays an essential role in the excellent lithium storage capacity, cyclability, and rate capability of this electrode.

In summary, particular N, S co-doped graphene/Fe_3_O_4_ architectures, consisting of uniformly dispersed Fe_3_O_4_ nanoparticles and N.S co-doped graphene encapsulant, have been successfully synthesized by a simple hydrothermal process, followed by a subsequent carbonization treatment. As one kind of potential anode material for lithium ion batteries, the N-S-G/Fe_3_O_4_ composite electrode exhibits a high initial reversible capacity of 1362.2 mAhg^−1^ with the coulombic efficiency of approximately 63.6% at the current density of 100 mAg^−1^. In addition, it delivers a high reversible specific capacity of over 854 mAhg^−1^ after the first 10 cycles, further maintains a reversible capacity of approximately 1055.20 mAhg^−1^ after 100 cycles with the coulombic efficiency of nearly 100%, much higher than that of the pure Fe_3_O_4_ electrode. The composite also exhibits good rate performance with specific capacity of 556.69 mAhg^−1^ when cycled at the current density of 1000 mAg^−1^. This particular composite architecture is characterized by having many meso/micro holes/pores surrounded by many randomly-aligned N, S co-doped graphene nanosheets. Those meso/micro holes/pores facilitate the lithium ion and electrolyte diffusion in these active materials during the charge/discharge processes. Furthermore, the essential N, S co-doped graphene nanosheets in the composite exert many important effects on encapsulating the Fe_3_O_4_ nanoparticles, greatly enhancing the conductivity of the active material, and increasing lithium intercalation sites owing to the high-level nitrogen and sulfur doping in the composite, as compared with the pure Fe_3_O_4_. Therefore, the composite is one of the most promising candidates as the potential anode material for LIBs, even though the composition and structure of those materials require further improvement.

## Methods

### Synthesis of N, S co-doped graphene/Fe_3_O_4_ nanostructures

N, S co-doped graphene/Fe_3_O_4_ nanostructures (N-S-G/Fe_3_O_4_) have been synthesized by a hydrothermal process, followed by a subsequent carbonization treatment. Firstly, the graphene oxide (**GO**) was obtained from oxidizing the graphite powder by a modified Hummers’method^56^, followed by two-hour sonication and centrifugation at the rate of 4000 rpm for 10 min to remove the aggregates and then1mg/ml GO solution was gained. Subsequently, 3.33 g dicyandiamide (99.9% Sigma-Aldrich), 336mg thiourea (99% Sigma-Aldrich), and 142.2 mg FeCl_2_·4H_2_O (Analytical Pure, Sinopharm Chemical Reagent Co. Ltd, China) were dispersed into 88 ml GO suspension. After stirring vigorously for 1 h, the resulting solution was transferred into a 100 ml Teflon-lined stainless steel autoclave, sealed and further heated at 150 °C for 12 h. After filtering, the solid products were obtained, and washed with deionized water several times to remove the remaining ions. The as-obtained dark grey products were dried at 60 °C in air for 12 h and then transferred to a ceramic crucible and carbonized at 500 °C for 2 h under nitrogen atmosphere. Finally, some dark N, S co-doped graphene/Fe_3_O_4_ powder was obtained and marked as N-S-G/Fe_3_O_4_. For comparison, the rGO/Fe_3_O_4_ was also prepared in the same condition in the absence of dicyandiamide and thiourea while the composites with different content of Fe_3_O_4_ were further synthesized in the same way via introducing different content of FeCl_2_·4H_2_O precursor (71.1 mg and 284.4 mg) during the synthesis procedure, marked as L-N-S-G/Fe_3_O_4_ and H-N-S-G/Fe_3_O_4_, respectively. Some Fe_3_O_4_ powder was simply prepared by the liquid precipitation reaction (**LPR**). Typically, 176 mg FeCl_3_∙6H_2_O and 130 mg FeCl_2_∙4H_2_O (Fe^3+^ and Fe^2+^ with a mole ratio of 1:1) were dispersed in 50 mL deionized water. After 1 M NaOH aqueous solution was dropped by drop added into the suspension until the pH ≈ 11, the mixture was stirred for 2 h at 80 °C in air. The dark products were cooled and washed several times with deionized water for several times and dried at 60 °C under vacuum environment.

### Materials Characterization

Thermogravimetric analysis (**TGA**) of the as-prepared N, S co-doped graphene/Fe_3_O_4_ nanocomposite was carried out with a TGA/DSC1 type instrument (TGA/DSC1 SF/1382, Mettler Switzerland, German) with a heating rate of 10 °C min^−1^ from room temperature to 1000 °C in air. The phase of the products was examined with an X’ Pert Pro MPD X-ray diffractometer with Cu Kα radiation (λ = 1.5418 Å, Philips, Holland). The morphology of these nanomaterials was evaluated with S-3000 scanning electron microscope (**SEM**, S-3000, HITACHI, Japan), NanoSEM 230 field emission scanning electron microscope (**FE-SEM**, Nova NanoSEM 230, FEI, America) and a Tecnai G2F20 S-TWIN transmission electron microscope (**TEM**, Tecnai GX F20 S-TWIN, FEI, America). The X-ray photoelectron spectroscopy (**XPS**) experiments were carried out on a VG Scientific ESCALAB 250 instrument (**XPS**, ESCALAB 250, Thermo Scientific, America) by using aluminum Kα X-ray radiation during XPS analysis. The Raman spectra were obtained on a Renishaw Invia Raman microscope excited by an argon ion laser beam (514.5 nm, 20 mW).

### Electrochemical Characterization

The electrochemical properties were further measured with electrodes that were prepared by compressing a mixture of the as-prepared active materials (Fe_3_O_4_ nanopowders or N, S co-doped graphene/Fe_3_O_4_ nanocomposites), carbon black (Super P, MMM, Belgium), and poly (vinyl difluoride) (PVDF) binder in a weight ratio of 70:15:15, and further coating the mixture onto one piece of copper foil. Pure lithium metal foil was used for the counter and reference electrode. The electrolyte was LiPF_6_ (1 M) in a mixture of ethylene carbonate (EC) and dimethyl carbonate (DMC) (1:1 v/v; MERCK KgaA, Germany). Coin cells were assembled in a high-purity argon-filled glove box. The galvanostatic method was utilized to measure the electrochemical capacity of the electrodes at room temperature on a LAND-CT2011A instrument with a charge–discharge current density of 100 mAg^−1^. Rate capability tests of the electrodes were then carried out systematically at various current densities (100 mAg^−1^, 200 mAg^−1^, 500 mAg^−1^, 1000 mAg^−1^). The cutoff potentials for charge and discharge in the cycling and rate tests were all set at 3.0 and 0.01 V versus Li^+^/Li, respectively. Cyclic-Voltammetry was performed on a CHI650D electrochemical workstation with the cutoff voltage range between 0.01–3.0 V. Electrochemical impedance spectroscopy (EIS) patterns were recorded using a CHI6500D electrochemical working station in the frequency range 100 kHz to 0.01 Hz with an amplitude of 10 mV.

## Additional Information

**How to cite this article**: Yang, Z. *et al.* Encapsulation of Fe_3_O_4_ Nanoparticles into N, S co-Doped Graphene Sheets with Greatly Enhanced Electrochemical Performance. *Sci. Rep.* **6**, 27957; doi: 10.1038/srep27957 (2016).

## Supplementary Material

Supplementary Information
