# Supplementary material for: Encapsulation of Fe3O4 Nanoparticles into N, S co-Doped Graphene Sheets with Greatly Enhanced Electrochemical Performance
Source: Sci Rep. 2016 Jun 14;6:27957. doi: 10.1038/srep27957 (PMC4906393; doi:10.1038/srep27957)
Supplement: Supplementary Information [file srep27957-s1.pdf]

# Encapsulation of $Fe_3O_4$ Nanoparticles into N, S co-Doped Graphene Sheets with Greatly Enhanced Electrochemical Performance

Zunxian Yang[1]*, Kun Qian[1], Jun Lv[1], Wenhuan Yan[1], Jiahui Liu[1], Jingwei Ai[1], Yuxiang Zhang[1], Tailiang Guo[1]*, Xiongtu Zhou[1], Sheng Xu[1], Zaiping Guo[2,3]*

[1] National & Local United Engineering Laboratory of Flat Panel Display Technology, Fuzhou University, Fuzhou 350002, P. R. China

[2]Institute for Superconducting & Electronic Materials, University of Wollongong, NSW 2522, Australia

[3]School of Mechanical, Materials & Mechatronics Engineering, University of Wollongong, NSW 2522, Australia

Correspondence and requests for materials should be addressed to Zunxian Yang (yangzunxian@hotmail.com), Zaiping Guo (zguo@uow.edu.au) or Tailiang Guo(gtlfzu@hotmail.com)

**Supporting Information**

**Captions**

**Figure S1** SEM and TEM images of graphene and as-prepared N-S-G/$Fe_3O_4$ composite: **(a)** SEM image of graphene; **(b)** SEM image of as-prepared N-S-G/$Fe_3O_4$ composite revealing there are many meso- or micro- holes surrounded by their nanosheets; **(c)** TEM image of as-prepared N-S-G/$Fe_3O_4$ composite indicating that there are also some large-size $Fe_3O_4$ particles distributed and encapsulated in the N, S co-doped graphene nanosheets; **(d)** high magnification TEM image of as-prepared N-S-G/$Fe_3O_4$ composite. **(e)** SEM image of pure $Fe_3O_4$; **(f)** higher magnification SEM image of $Fe_3O_4$ particles revealing there are many large-size particles.

**Figure S2** Elemental mapping images of N-S-G/$Fe_3O_4$ composite. **(a)** Typical scanning electron microscopy (STEM), **(b)** STEM image taken from the square region in **(a)**; **(c)** Elemental mapping image of carbon; **(d)** Elemental mapping image of oxygen; **(e)** Elemental mapping image of nitrogen; **(f)** Elemental mapping image of iron;. **(g)** Elemental mapping image of sulfur; **(h)** the energy dispersive spectroscopy (EDS) of N-G/$Fe_3O_4$ composite (**inset** reports the content of all the elements in the N-S-G/$Fe_3O_4$ composite and the content of the $Fe_3O_4$ calculated from that of the iron element).

**Figure S3** Thermogravimetric (**TGA**) and different scanning calorimetric (**DSC**) analysis of N-S-G/$Fe_3O_4$ composite.

**Figure S4** Electrochemical performance of $Fe_3O_4$ nanoparticles, rGO/ $Fe_3O_4$, L-N-S-G/$Fe_3O_4$ and H-N-S-G/$Fe_3O_4$ composite electrodes cycled between 0.01 and

3.0 V vs. $Li^+$/Li: **(a)**, **(b)** Cyclic voltammograms of $Fe_3O_4$ nanoparticles and rGO/ $Fe_3O_4$ composite electrode from the first cycle to the fifth cycle at a scan rate of 0.1 $mVs^{-1}$ in the voltage range of 0.01-3.0 V. **(c)**, **(d)** Voltage profiles for selected cycles of $Fe_3O_4$ nanoparticles and rGO/ $Fe_3O_4$ composite electrode at the current density of 100 $mAg^{-1}$. **(e)**, **(f)** Capacity vs. cycle number curves and coulombic efficiency from the first cycle to the $100^{th}$ cycle for the rGO/ $Fe_3O_4$, L-N-S-G/$Fe_3O_4$ and H-N-S-G/$Fe_3O_4$ composite at the current density of 100 $mAg^{-1}$ with cut-off voltage between 0.01 and 3.0V. **(g)**, **(h)** Rate capabilities of rGO/ $Fe_3O_4$, L-N-S-G/$Fe_3O_4$ and H-N-S-G/$Fe_3O_4$ composite electrodes at various currents (100 mA/g, 200 mA/g, 500 mA/g, 1000 mA/g).

**Figure S5** Nyquist plots of N-G/$Fe_3O_4$ and $Fe_3O_4$ particle electrodes (Note: the **insets** indicate the simulation equivalent circuits, the parameters of elements and the fitting profiles)

**Figure S6** the lithiation / delithiation scheme of the N-S-G / $Fe_3O_4$ composites indicating the paths for lithium-ion and electrons

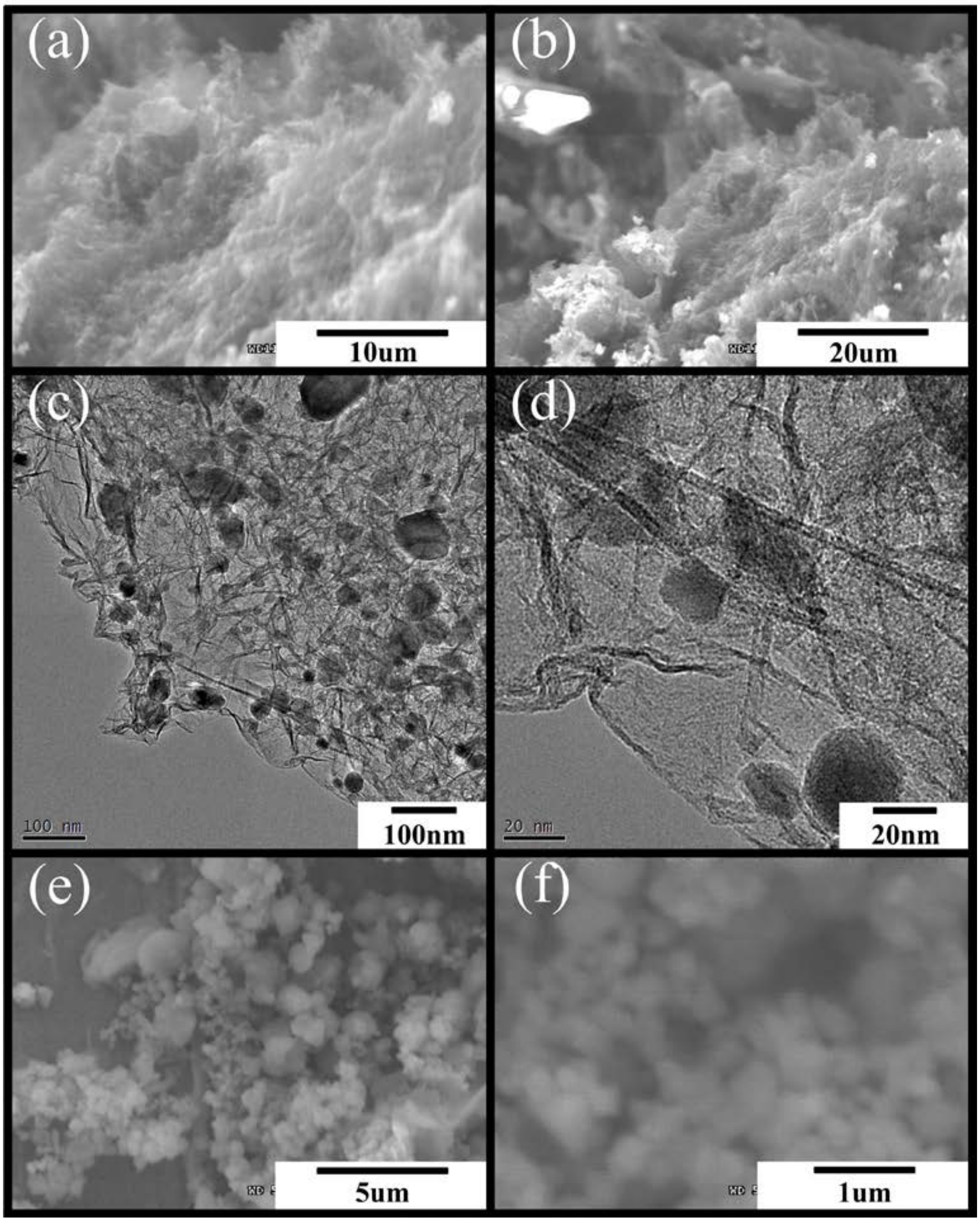


**Figure S1**

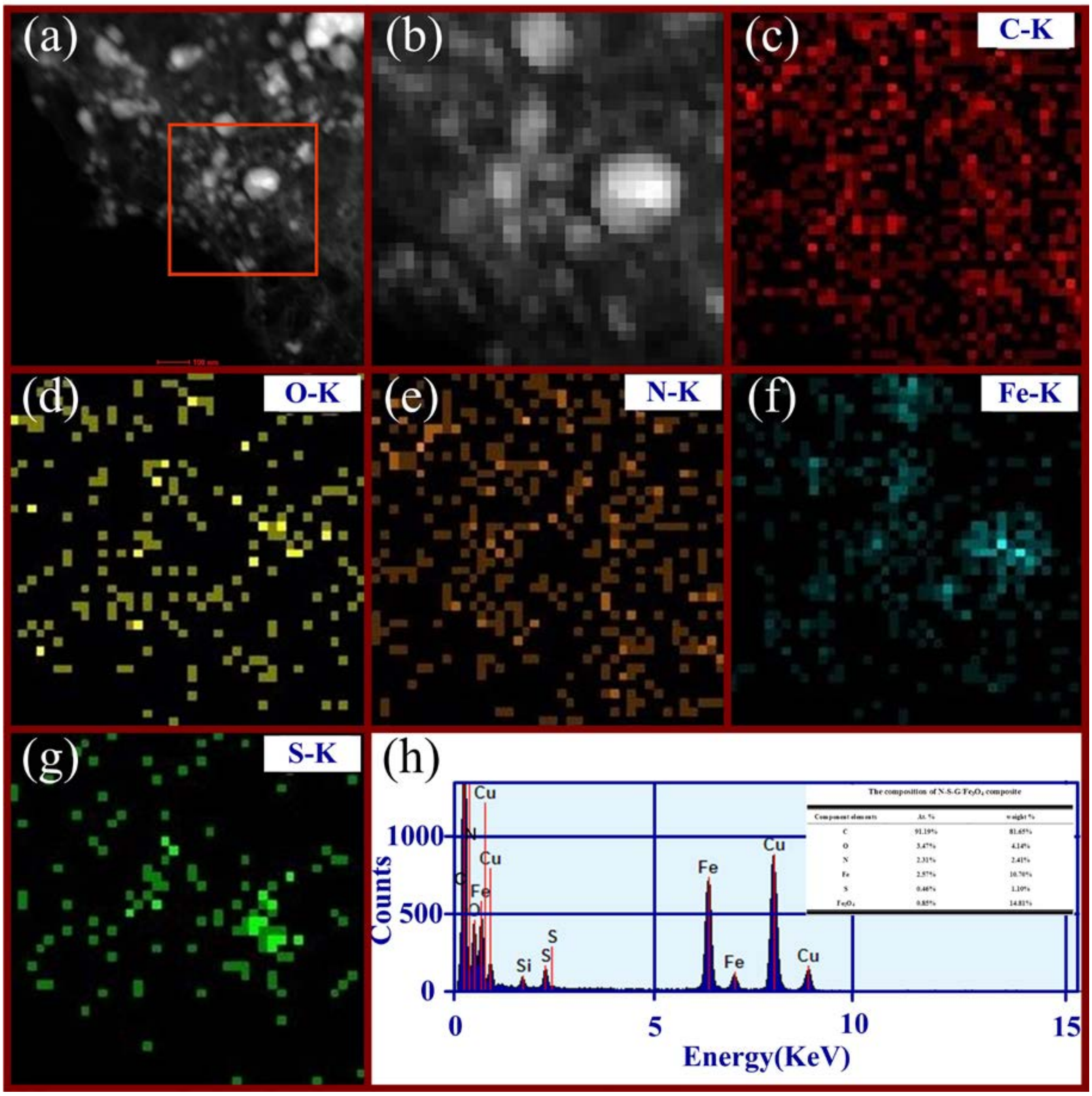
(a)
(b)
(c)
C-K
(d)
O-K
(e)
N-K
(f)
Fe-K
(g)
S-K
(h)
Counts
Energy(KeV)
0
5
10
15
500
1000
Cu
N
C
Fe
O
Si
S
The composition of N-S-G-$Fe_3O_4$ composite
Component elements | At. % | weight %
C | 91.19% | 81.65%
O | 3.47% | 4.14%
N | 2.31% | 2.41%
Fe | 2.57% | 10.70%
S | 0.46% | 1.10%
$Fe_3O_4$ | 0.85% | 14.81%

**Figure S2**

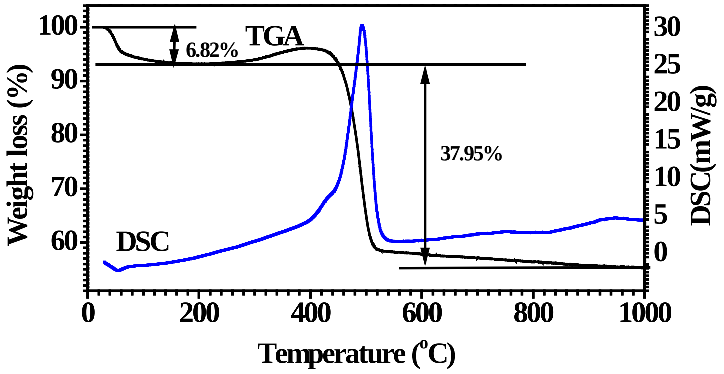
TGA
6.82%
37.95%
DSC
Weight loss (%)
DSC(mW/g)
Temperature (°C)
100
90
80
70
60
30
25
20
15
10
5
0
200
400
600
800
1000

**Figure S3**

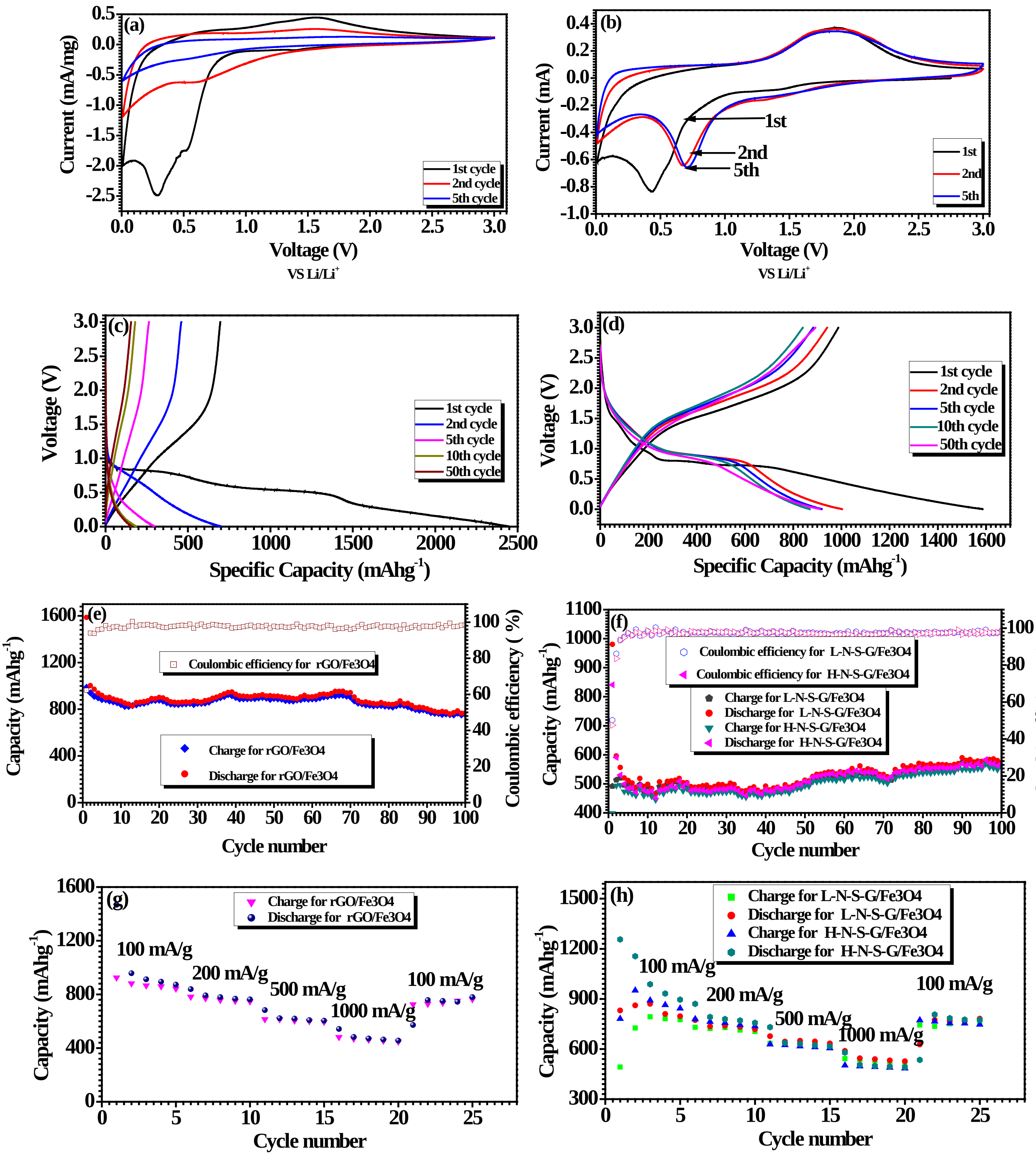
(a)
Current (mA/mg)
Voltage (V)
VS Li/Li+
1st cycle
2nd cycle
5th cycle
(b)
Current (mA)
1st
2nd
5th
(c)
Voltage (V)
Specific Capacity (mAhg-1)
1st cycle
2nd cycle
5th cycle
10th cycle
50th cycle
(d)
(e)
Capacity (mAhg-1)
Coulombic efficiency ( %)
Coulombic efficiency for rGO/Fe3O4
Charge for rGO/Fe3O4
Discharge for rGO/Fe3O4
Cycle number
(f)
Coulombic efficiency for L-N-S-G/Fe3O4
Coulombic efficiency for H-N-S-G/Fe3O4
Charge for L-N-S-G/Fe3O4
Discharge for L-N-S-G/Fe3O4
Charge for H-N-S-G/Fe3O4
Discharge for H-N-S-G/Fe3O4
(g)
100 mA/g
200 mA/g
500 mA/g
1000 mA/g
(h)

**Figure S4**

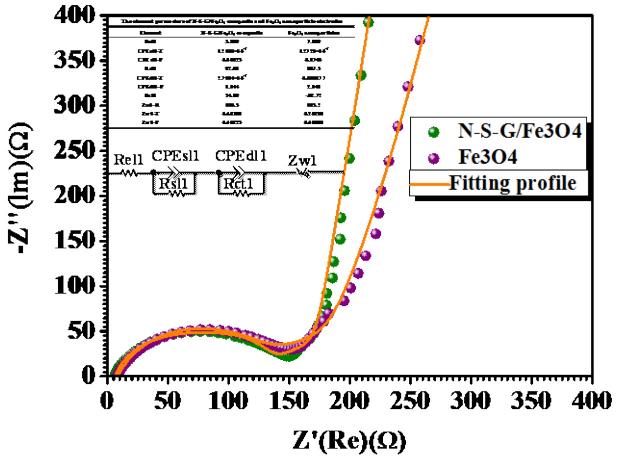

-Z''(Im)(Ω)
Z'(Re)(Ω)
0
50
100
150
200
250
300
350
400
Rel1
CPEsl1
CPEdl1
Zw1
Rsl1
Rct1
N-S-G/Fe3O4
Fe3O4
Fitting profile


**Figure S5**

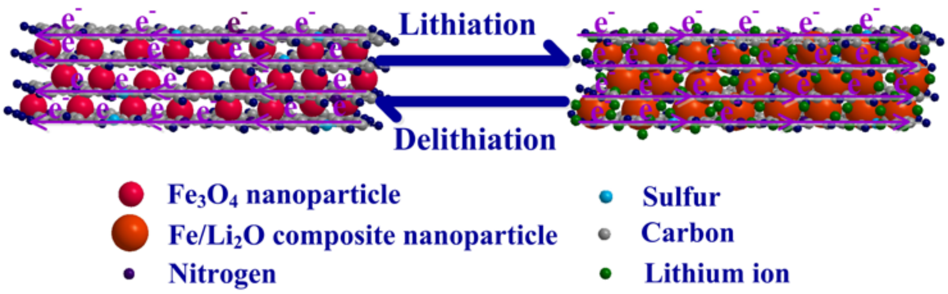
Lithiation
Delithiation
$Fe_3O_4$ nanoparticle
$Fe/Li_2O$ composite nanoparticle
Nitrogen
Sulfur
Carbon
Lithium ion

**Figure S6**
